# A new species of *Trismegistomya* Reinhard (Diptera: Tachinidae) from Area de Conservación Guanacaste in northwestern Costa Rica

**DOI:** 10.3897/BDJ.7.e29130

**Published:** 2019-04-10

**Authors:** AJ Fleming, D. Monty Wood, M. Alex Smith, Tanya Dapkey, Winnie Hallwachs, Daniel H Janzen

**Affiliations:** 1 Agriculture Agri-Food Canada, Ottawa, Canada Agriculture Agri-Food Canada Ottawa Canada; 2 Department of Integrative Biology and the Biodiversity Institute of Ontario, Guelph, Canada Department of Integrative Biology and the Biodiversity Institute of Ontario Guelph Canada; 3 Department of Biology, University of Pennsylvania, Philadelphia, United States of America Department of Biology, University of Pennsylvania Philadelphia United States of America

**Keywords:** Dexiinae, Voriini, tropical rain forest, tropical dry forest, parasitoid flies, Erebidae, host specificity, Guanacaste, Caterpillar

## Abstract

**Background:**

The New World genus *Trismegistomya* Reinhard, 1967b (Diptera: Tachinidae) previously included only the type species *Trismegistomya pumilis* (Reinhard, 1967a) from Arizona, U.S.A.

**New information:**

We describe a new species of *Trismegistomya*, *Trismegistomya jimoharai* Fleming & Wood **sp. n.**, from Area de Conservación Guanacaste (ACG) in northwestern Costa Rica, reared from wild-caught caterpillars of *Melipotis januaris* (Guenée, 1852) (Lepidoptera: Erebidae). Our study provides a concise description of the new species using morphology, life history, molecular data and photographic documentation. In addition to the new species description, we provide a redescription of the genus, as well as of its type species *Trismegistomya pumilis*.

## Introduction

The monotypic genus *Trismegistomya* Reinhard, 1967b (Dexiinae: Voriini) was initially erected and described under the name *Trismegistus* Reinhard, 1967a from a single female specimen collected in Portal, Arizona; however, as this name was preoccupied by *Trismegistus* Johnson & Snyder, 1904, it was replaced in that same year by *Trismegistomya* Reinhard, 1967b. In his original description, the author compared the type species, *Trismegistus pumilis* Reinhard, 1967a, to *Myiophasia* Brauer & Bergenstamm, 1891 (a parasitoid of Coleoptera and now a synonym of *Gnadochaeta* Macquart, 1850), but mentioned that they had "decisively different cephalic characters."

*Trismegistomya* belongs to the tribe Voriini within the subfamily Dexiinae (O'Hara and Wood 2004). The Voriini are a cosmopolitan assemblage of genera, with a strong representation in the Neotropics. Voriini are a quite well-studied tribe; one of the more recent papers, on the Voriini of Chile, provided information on their biology, hosts and distribution (Cortés and González 1989). The tribe can generally be characterized by the following combination of character states: conical head profile (longer at level of pedicel than at vibrissa); proclinate, divergent and well developed ocellar setae; frons wide; proclinate and reclinate orbital setae present in both sexes; facial ridge bare; prosternum bare; anepimeral seta absent or poorly developed so as to appear hair-like; infrasquamal setae present; apical scutellar setae strong and decussate; dm-cu crossvein oblique, making posterior section of CuA_1_ equal to anterior section; R_4+5_ setulose at least to crossvein r-m and sometimes beyond; middorsal depression of ST1+2 reaching posterior margin; and aedeagus elongate and frequently ribbon-like (Cortés and González 1989). Voriini parasitize larvae of Lepidoptera, primarily belonging to families of Noctuoidea (Guimarães 1977), by laying flattened membranous incubated eggs directly on the cuticle of the host (Herting 1957).

To date, there has been no other work on *Trismegistomya* since its original description. This work aims to build on the knowledge of the genus by adding a new species based on differences in external morphology and by providing COI (coxI or cytochrome *c* oxidase I) gene sequences. We also add a description of the previously unknown male of *Trismegistomya pumilis* (Reinhard, 1967b). This paper is part of a broader effort to name and catalog all of the tachinid species collected from the ACG inventory (Fleming et al. 2014, Fleming et al. 2014, Fleming et al. 2015b, Fleming et al. 2015c, Fleming et al. 2015a, Fleming et al. 2015, Fleming et al. 2016a, Fleming et al. 2016b, Fleming et al. 2017). This series of taxonomic papers will represent a foundation for later, detailed ecological and behavioral accounts and studies extending across ACG ecological groups, whole ecosystems and taxonomic assemblages much larger than a genus.

## Materials and methods

### Project aims and rearing intensity

All reared specimens were obtained from host caterpillars collected in Area de Conservación Guanacaste (ACG) (Janzen et al. 2009, Janzen et al. 2011, Janzen and Hallwachs 2011, Fernandez-Triana et al. 2014, Janzen and Hallwachs 2016). ACG's 125,000+ terrestrial hectares cover portions of the provinces of Alajuela and Guanacaste inclusive of the dry forested northwestern coast of Costa Rica and, inland, of the Caribbean lowland rainforest. ACG comprises three different ecosystems and their intergrades, ranging from sea level to 2000 m. The tachinid rearing methods are described at http://janzen.bio.upenn.edu/caterpillars/methodology/how/parasitoid_husbandry.htm. Since its inception, this inventory has reared over 750,000 wild-caught ACG caterpillars. Any frequencies of parasitism reported here need to be considered against this background inventory (Smith et al. 2005, Smith et al. 2006, Smith et al. 2007, Smith et al. 2008, Janzen et al. 2009, Smith et al. 2009, Janzen et al. 2011, Janzen and Hallwachs 2011, Rodriguez et al. 2012, Smith et al. 2012, Janzen and Hallwachs 2016). Comparative details of the parasitoid ecology of these flies will be treated separately in later papers, once the alpha-taxonomy of ACG caterpillar-attacking tachinids is more complete.

### Voucher specimen management

Voucher specimen management follows the methods first outlined in Fleming et al. (2014). All caterpillars reared from the ACG efforts receive a unique voucher code in the format yy–SRNP–xxxxx. Any parasitoid emerging from a caterpillar receives the same voucher code as a record of the rearing event. If and when the parasitoid is later dealt with individually it receives a second voucher code unique to it, in the format DHJPARxxxxxxx. These voucher codes assigned to both host and parasitoids may be used to obtain the individual rearing record at http://janzen.bio.upenn.edu/caterpillars/database.lasso.

To date, all DHJPARxxxxxx-coded tachinids have had one leg removed for DNA barcoding at the Biodiversity Institute of Ontario (BIO) in Guelph, ON, Canada. All successful barcodes and collateral data are first deposited in the Barcode of Life Data System (BOLD, www.boldsystems.org) (Ratnasingham and Hebert 2007) and later migrated to GenBank. Each barcoded specimen is also assigned unique accession codes from the Barcode of Life Data System (BOLD) and GenBank, respectively.

Inventoried Tachinidae were collected under Costa Rican government research permits issued to DHJ and exported from Costa Rica to Philadelphia, en route to their final depository in the Canadian National Insect collection in Ottawa, Canada (CNC). Tachinid identifications for the inventory were conducted by DHJ in coordination with a) morphological inspection by AJF and DMW, b) DNA barcode sequence examination by MAS and DHJ and c) correlation with host caterpillar identifications by DHJ and WH through the inventory itself. Dates of collection cited for each ACG specimen are the dates of eclosion of the fly, not the date of capture of the caterpillar, since the fly eclosion date is much more representative of the time when that fly species is on the wing rather than the time of capture of the host caterpillar. The collector listed on the label is the parataxonomist who found the caterpillar, rather than the person who retrieved the newly eclosed fly from its rearing container. The holotypes of the species newly described herein are all deposited at CNC.

### Acronyms for depositories

CNC Canadian National Collection of Insects, Arachnids and Nematodes, Ottawa, Canada

### Descriptions and imaging

The description of the new species presented here is complemented with a series of color photos, used to illustrate the morphological differences with already known species. Imaging was carried out using the methods outlined in Fleming et al. (2014). The morphological terminology used follows Cumming and Wood (2009). Measurements and examples of anatomical landmarks discussed herein are illustrated in Fig. 1. Male terminalia were not examined, as the material was scarce.

### DNA Barcoding

The DNA barcode region (5’ cytochrome *c* oxidase I (CO1) gene, Hebert et al. 2003) was examined from two specimens of ACG *Trismegistomya jimoharai* **sp. n.** We obtained DNA extracts from a single leg using a standard glass fiber protocol (Ivanova et al. 2006). We amplified the 658 bp region near the 5’ terminus of the CO1 gene using standard primers (LepF1–LepR1), following established protocols for production and quality control (Smith et al. 2006, Smith et al. 2007, Smith et al. 2008). Interested readers may consult the Barcode of Life Data System (BOLD) (Ratnasingham and Hebert 2007) for information associated with each sequence (including GenBank accessions), using the persistent DOI: dx.doi.org/10.5883/DS-ASTRIS.

## Taxon treatments

### Trismegistomya

Reinhard, 1967

Trismegistomya Reinhard, 1967 - Reinhard 1967b:600, *nomen novum* for *Trismegistus* Reinhard, 1967.
Trismegistus Reinhard, 1967 - Reinhard 1967a:100, junior homonym of *Trismegistus* Johnson & Snyder, 1904.
Trismegistomya Trismegistus pumilis Reinhard, 1967Reinhard 1967a: 100. by original designation

#### Description

Male. **Head**: strongly conical in shape, 1.3× wider than tall in frontal view, in profile 1.35× wider at level of pedicel than at level of vibrissa; fronto-orbital plate and parafacial dull grey-silver tomentose; frontal vitta of reddish-brown color, 1.2× width of fronto-orbital plate; eye bare, occupying 0.75× height of head; postpedicel 1.18× pedicel; pedicel brilliant orange; arista slightly longer than postpedicel, abruptly tapered apically; antennal insertion level with middle of eye; gena almost 0.25× height of eye, strong genal groove of deep dull red color, contrasting with silver gena; two pairs of vertical setae, inner vertical setae convergent, almost 2x as long as outer vertical setae, which are strongly divergent; ocellar setae weak but present and strongly divergent; fronto-orbital plate with single row of frontal setae, at most one frontal seta below upper margin of pedicel and two rows of short setulae outside of frontal setae; parafacial bearing one row of weak proclinate parafacial setae directly adjacent to facial ridge (so close that the facial ridge appears as setulose), appearing as a continuation of frontal setae; one row of scattered setulae on remainder of parafacial, extending to lower proclinate orbital seta; two pairs of proclinate orbital setae, one pair of lateraloclinate or reclinate upper orbital setae; palpus yellow-orange, only slightly haired. **Thorax**: entirely black; dorsally with a very slight grey tomentum presuturally, only visible under certain angles of light, otherwise appearing as glabrous black; chaetotaxy: three postpronotal setae arranged in a straight line; two notopleural setae; three postsutural acrostichal setae; 3–4 postsutural dorsocentral setae; 2–3 postsutural intra-alar setae; three postsutural supra-alar setae; 2–3 katepisternal setae; anepimeron bare, with 2–4 short and stout hair-like setae, lacking any strong elongate anepimeral setae; scutellum with 1–2 pairs oflateral setae; one pair of apical setae; discal scutellar setae ranging from one pair to absent. **Wings**: hyaline with a slight yellow tinge; bend of vein M obtuse, ending at wing margin; crossvein dm-cu slightly oblique; wing vein R_4+5_ bearing 4–6 setulae dorsally, extending 0.75× distance from node to crossvein r-m. **Legs**: short and stout; entirely glabrous black, densely covered in appressed short setulae. **Abdomen**: ground color appearing glabrous reddish-black; very slight silver tomentum visible on anterior margins of tergites when viewed under different angles of light; mid-dorsal depression of ST1+2 extending only halfway across syntergite, not reaching tergal margin; marginals present only as a complete row of setae on both T4 and T5; one row of discal setae on T5. Females differ from males only in their terminalia.

#### Diagnosis

*Trismegistomya* is distinguished from other voriines by the following combination of characters: small, 3–3.5 mm long; habitus glabrous black, with only light tomentum presuturally only evident under certain angles of light; conical head profile with axis of pedicel subequal to head height; deeply excavated clypeus; vibrissa inserted above lower margin of face; yellowish wing reaching beyond tip of abdomen; abdomen ovate with mid-dorsal depression of ST1+2 not reaching tergal margin, possessing median marginal setae on T4 and T5 and median discal setae on T5 only.

#### Distribution

New World genus ranging from Arizona in the USA south to Costa Rica.

### Trismegistomya jimoharai

Fleming & Wood sp. n.

urn:lsid:zoobank.org:act:790573DE-5676-4D55-B62B-78CDBDE101C7

#### Materials

- **Type status:** Holotype. **Occurrence:** occurrenceDetails: http://janzen.sas.upenn.edu; catalogNumber: DHJPAR0018620; recordedBy: D.H. Janzen & W. Hallwachs; individualID: DHJPAR0018620; individualCount: 1; sex: male; lifeStage: adult; preparations: pinned; otherCatalogNumbers: 84-SRNP-154.3,BOLD:AAW7939,ASTAI1267-07; **Taxon:** scientificName: Trismegistomya jimoharai; phylum: Arthropoda; class: Insecta; order: Diptera; family: Tachinidae; genus: Trismegistomya; specificEpithet: jimoharai; scientificNameAuthorship: Fleming & Wood, 2015; **Location:** continent: Central America; country: Costa Rica; countryCode: CR; stateProvince: Guanacaste; county: Sector Santa Rosa; locality: Area de Conservación Guanacaste; verbatimLocality: Vado Nisperal; verbatimElevation: 10; verbatimLatitude: 10.802; verbatimLongitude: -85.654; verbatimCoordinateSystem: Decimal; decimalLatitude: 10.802; decimalLongitude: -85.654; **Identification:** identifiedBy: AJ Fleming; dateIdentified: 2015; **Event:** samplingProtocol: Reared from Erebidae moth larva, *Melipotis januaris*; verbatimEventDate: 05/25/1984; **Record Level:** language: en; institutionCode: CNC; collectionCode: Insects; basisOfRecord: Pinned Specimen
- **Type status:** Paratype. **Occurrence:** occurrenceDetails: http://janzen.sas.upenn.edu; catalogNumber: DHJPAR0018621; recordedBy: D.H. Janzen & W. Hallwachs; individualID: DHJPAR0018621; individualCount: 1; sex: male; lifeStage: adult; preparations: pinned; otherCatalogNumbers: 84-SRNP-154,ASTAI1268-07; **Taxon:** scientificName: Trismegistomya jimoharai; phylum: Arthropoda; class: Insecta; order: Diptera; family: Tachinidae; genus: Trismegistomya; specificEpithet: jimoharai; scientificNameAuthorship: Fleming & Wood, 2015; **Location:** continent: Central America; country: Costa Rica; countryCode: CR; stateProvince: Guanacaste; county: Sector Santa Rosa; locality: Area de Conservación Guanacaste; verbatimLocality: Vado Nisperal; verbatimElevation: 10; verbatimLatitude: 10.802; verbatimLongitude: -85.654; verbatimCoordinateSystem: Decimal; decimalLatitude: 10.802; decimalLongitude: -85.654; **Identification:** identifiedBy: AJ Fleming; dateIdentified: 2015; **Event:** samplingProtocol: Reared from Erebidae moth larva, *Melipotis januaris*; verbatimEventDate: 05/25/1984; **Record Level:** language: en; institutionCode: CNC; collectionCode: Insects; basisOfRecord: Pinned Specimen

#### Description

**Male** (Fig. 2). Length: 3 mm. **Head** (Fig. 2b): postpedicel orange basally, directly adjacent to pedicel; arista orange on apical half; ocellar setae well-developed, proclinate and divergent, arising behind anterior ocellus; fronto-orbital plate with two regular rows of short setulae outside of frontal setae; frontal setae appearing as continuous with parafacial setulae; two pairs of proclinate orbital setae, one pair of reclinate upper orbital setae. **Thorax** (Fig. 2a, c): three postsutural supra-alar setae, middle seta twice as long as outer two; two postsutural intra-alar setae; four postsutural dorsocentral setae; scutellum with one pair of basal scutellar setae, two pairs of lateral scutellars and one pair of weaker divergent apical scutellar setae; scutellum bearing one pair of weak but evident discal setae slightly wider than apical scutellars. Legs (Fig. 2c): entirely glabrous black, as in generic description. Wing (Fig. 2a): hyaline, very slightly darkening basally; basicosta dark brownr; wing vein R_4+5_ bearing 5–6 setulae extending 0.75× distance from node to crossvein r-m on dorsal surface; ventral surface bearing at most 0–2 setulae; calypters pale white translucent. **Abdomen** (Fig. 2a, c): ground color of abdomen glabrous maroon or reddish-black; very slight silver tomentum only visible when observed under different angles of light, appearing as a silver sheen on dorsal surface of tergites; complete row of marginal setae present on both T4 and T5; median discal setae absent on all tergites except row on T5.

**Female**: unknown at this time, assumed to be similar to male as is the case with the type species.

#### Diagnosis

*Trismegistomya jimoharai* **sp. n.** is easily differentiated from its only congener, the type species *T. pumilis* Reinhard, by the following combination of traits: basal portion of postpedicel distinctly orange; arista orange apically; and four postsutural dorsocentral setae.

#### Etymology

*Trismegistomya jimoharai* **sp. n.** is named in honor of Dr. James O’Hara of Ottawa, Canada in recognition of his many years of support to the curation, taxonomy and administrative logistics of the on-going effort to inventory the caterpillar-attacking species of Tachinidae of ACG and secure the residence of their voucher specimens in the Canadian National Collection.

#### Distribution

Costa Rica, ACG, Prov. Guanacaste, coastal margin dry forest, 10 m elevation.

#### Ecology

*T. jimoharai* has been reared two times, from only four larvae of *Melipotis januaris* (Guenée, 1852) (Lepidoptera: Erebidae) reared by the inventory to date and collected while feeding on *Pithecellobium oblongum* Benth. (Fabaceae) in ACG dry forest. It should be noted that *Trismegistomya* has not been noted out of the other 808 rearings of *Melipotis* spp. reared by the inventory.

### Trismegistomya pumilis

(Reinhard, 1967)

Trismegistus pumilis Reinhard, 1967: 101. Type data: Holotype ♀ (CNC). U.S.A., Arizona, Portal. **Type label**: Holotype ♀: “Porta, Arz. VIII–17–65/ H J Reinhard Collector/ HOLOTYPE *Trismegistomya pumilis*/ *Trismegistomya pumulis* [sic] R (RNH).”

#### Materials

- **Type status:** Holotype. **Occurrence:** sex: female; **Taxon:** scientificName: *Trismegistomyia pumilis* (Reinhard, 1967); originalNameUsage: *Trismegistus pumilis* Reinhard, 1967; genus: Trismegistomyia; specificEpithet: pumilis; **Location:** locationID: Portal, Arizona; country: United States; countryCode: US; stateProvince: Arizona; locality: Portal; verbatimLocality: Portal, Arizona, USA; **Event:** year: 1965; month: 8; day: 17; verbatimEventDate: VIII–17–65; habitat: Desert; **Record Level:** institutionCode: CNC
- **Type status:** Other material. **Occurrence:** recordedBy: J.E. O'Hara; individualCount: 3; sex: 1 male, 2 females; lifeStage: adult; **Taxon:** scientificName: Trismegistomya pumilis; **Location:** countryCode: USA; stateProvince: Arizona; county: Graham County; locality: 2.4mi W on hwy 366 from hwy 191, 3800 ft.; verbatimLocality: 2.4mi W on hwy 366 from hwy 191; verbatimElevation: 3800ft; **Event:** samplingProtocol: Malaise trap; eventDate: 19–22.viii.1993; **Record Level:** institutionID: CNC; institutionCode: CNC

#### Description

**Female** (Fig. 3). Length: 3–4 mm. **Head** (Fig. 3b): pedicel bright orange; postpedicel dark brown throughout; arista concolorous with postpedicel, abruptly tapering apically; ocellar setae well-developed, proclinate and divergent, arising behind anterior ocellus; fronto-orbital plate with two irregular rows of short setulae outside of frontal setae; frontal setae appearing as continuous with parafacial setulae; two pairs of proclinate orbital setae, one pair of reclinate upper orbital setae. **Thorax** (Fig. 3a, c): three postsutural supra-alar setae (anteriormost seta weak, nearly half as long other two), three postsutural intra-alar setae; three postsutural dorsocentral setae; scutellum with one pair of basal scutellar setae, one pair of lateral scutellar setae and one pair of weaker divergent apical scutellar setae; scutellum bearing one pair of weak but evident discal setae slightly more widely separated than apical scutellar setae. Legs (Fig. 3c) entirely glabrous black, as in generic description. Wing (Fig. 3a): transparent with slight yellow tinge basally; basicosta of slightly burnt orange color; wing vein R_4+5_ bearing 5–6 setulae extending 0.75× distance from node to crossvein r-m on dorsal surface, ventral surface bearing 0–2 setulae; calypters pale white translucent, slightly cream colored basally. **Abdomen** (Fig. 3a, c): ground color of abdomen glabrous maroon or reddish-black; very slight silver tomentum only visible when observed under different angles of light, almost absent; complete row of marginal setae present on both T4 and T5; median discal setae absent on all tergites except row on T5.

**Male**: as female, except for terminalia.

#### Diagnosis

*Trismegistomyia pumilis* can be differentiated from its only congener, *T. jimoharai* **sp. n.**, by the following distinctive combination of traits: basal portion of postpedicel not distinctly orange; arista concolorous with postpedicel; and three postsutural dorsocentral setae instead of four.

#### Distribution

USA, Arizona, Portal.

#### Ecology

Unknown; specimens of *T. pumilis* were collected via Malaise traps and sweeping.

## Identification Keys

### Key to the species of *Trismegistomya* Reinhard

**Table:** 

1	Postpedicel dark brown throughout; arista concolorous with postpedicel; three postsutural dorsocentral setae.	*Trismegistomya pumilis* Reinhard
–	Basal portion of postpedicel distinctly orange; arista orange apically; four postsutural dorsocentral setae.	*Trismegistomya jimoharai* **sp. n.**

## Supplementary Material

XML Treatment for Trismegistomya

XML Treatment for Trismegistomya jimoharai

XML Treatment for Trismegistomya pumilis

## Figures and Tables

**Figure 1a.**
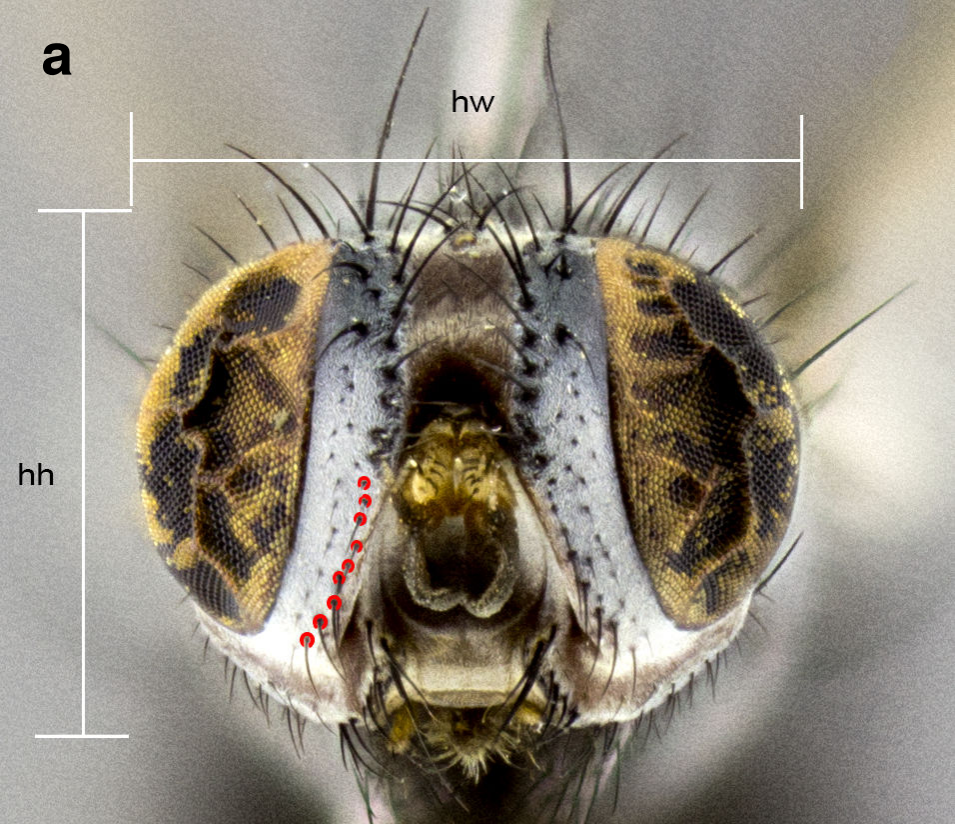
Sample of measured areas from front of head as shown on *Trismegistomya jimoharai* **sp. n.**; proclinate parafacial setae highlighted red; abbreviations: hh, head height; hw, head width.

**Figure 1b.**
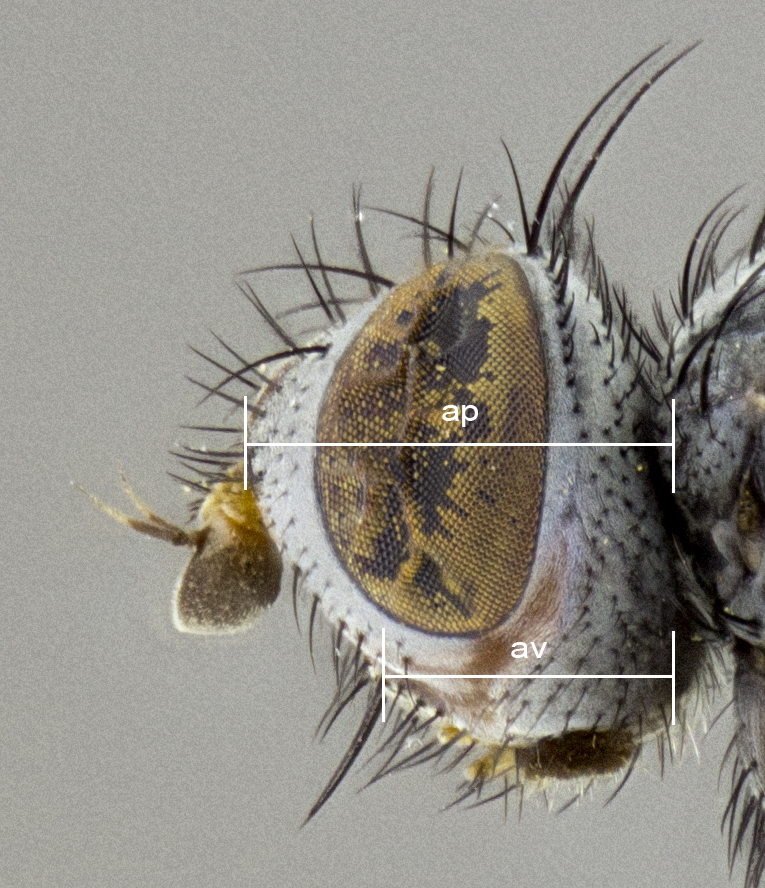
Sample of measured areas from side of head as shown on *Trismegistomya jimoharai* **sp. n.**; ap, axis of pedicel; av, axis of vibrissa.

**Figure 2a.**
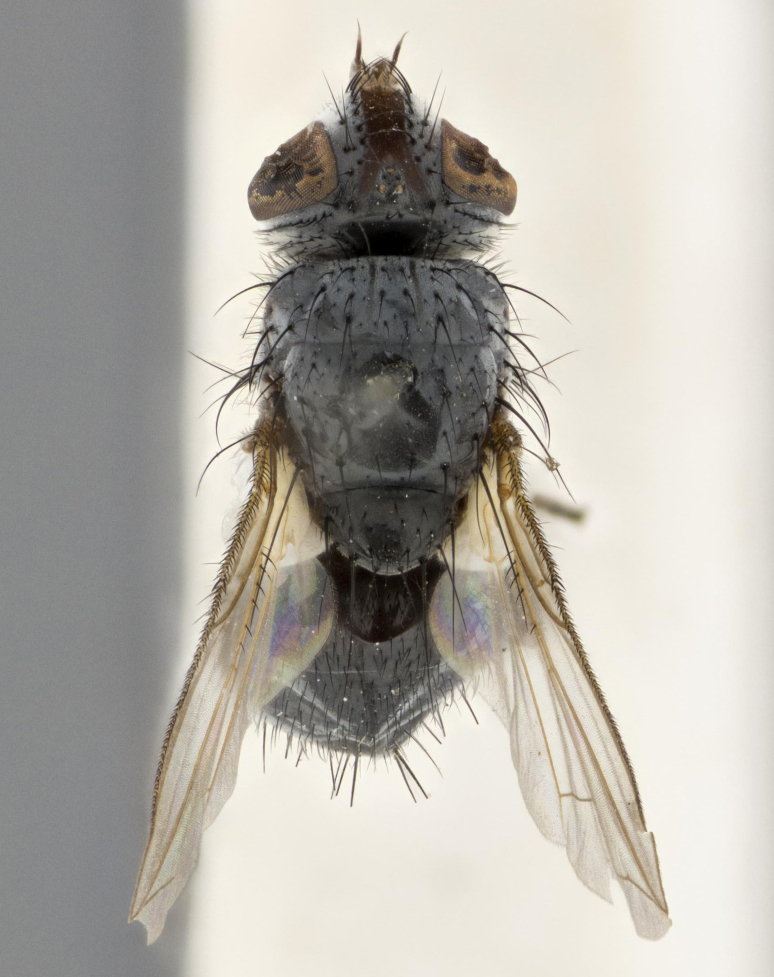
dorsal view

**Figure 2b.**
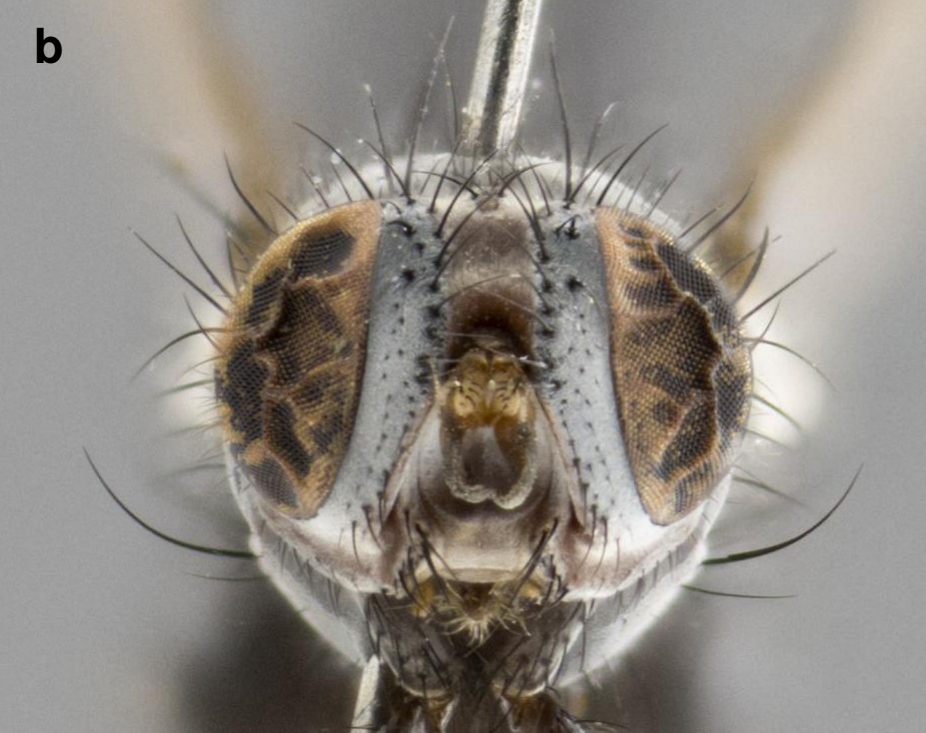
frontal view

**Figure 2c.**
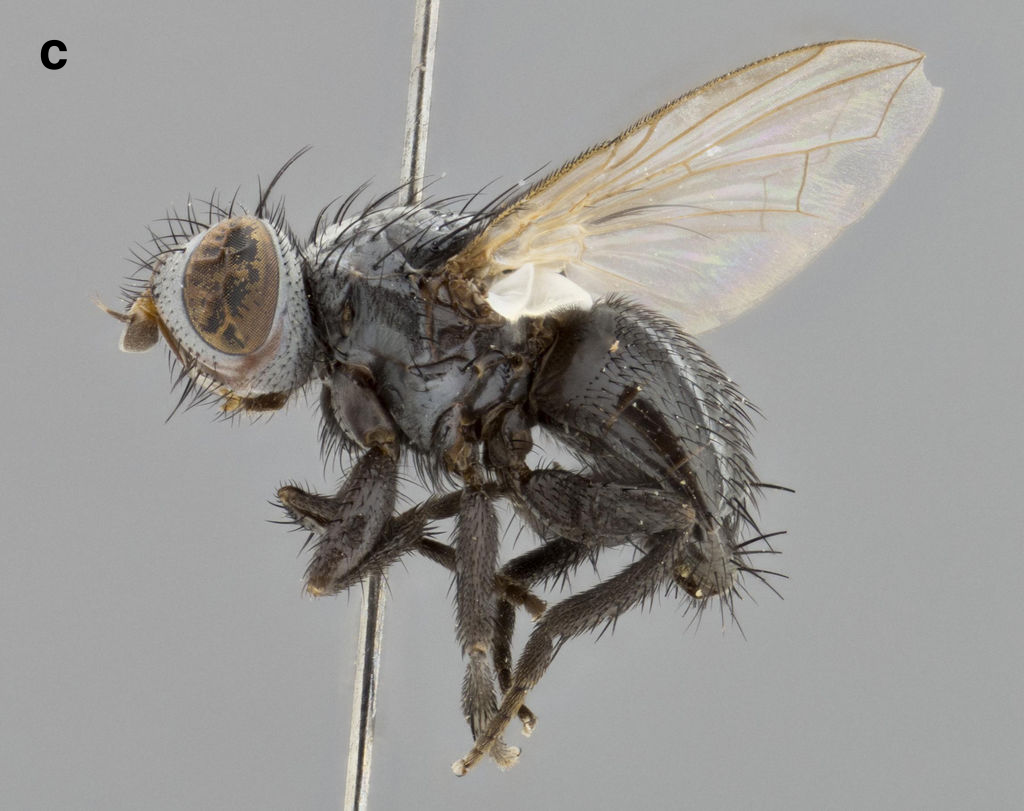
lateral view

**Figure 3a.**
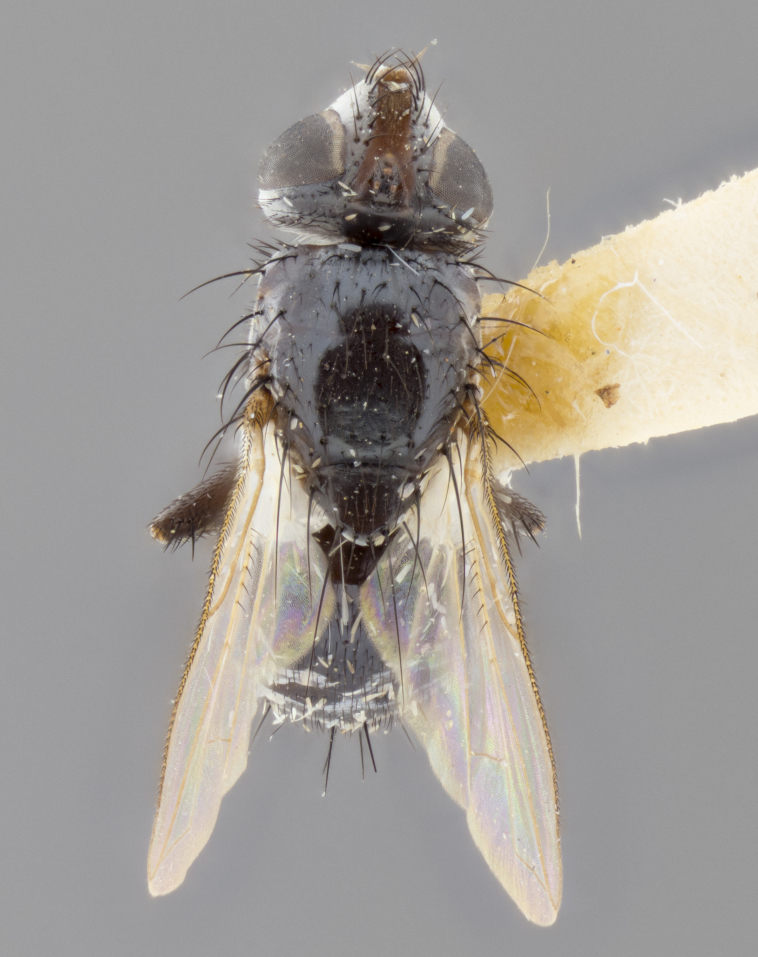
dorsal view

**Figure 3b.**
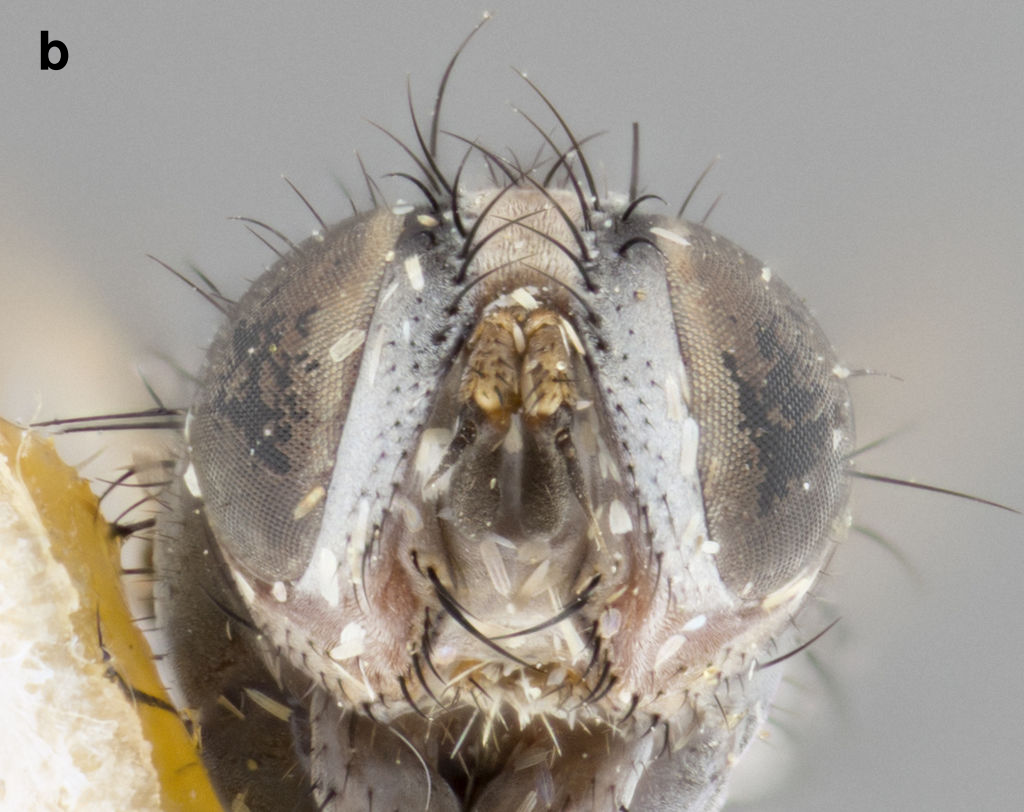
frontal view

**Figure 3c.**
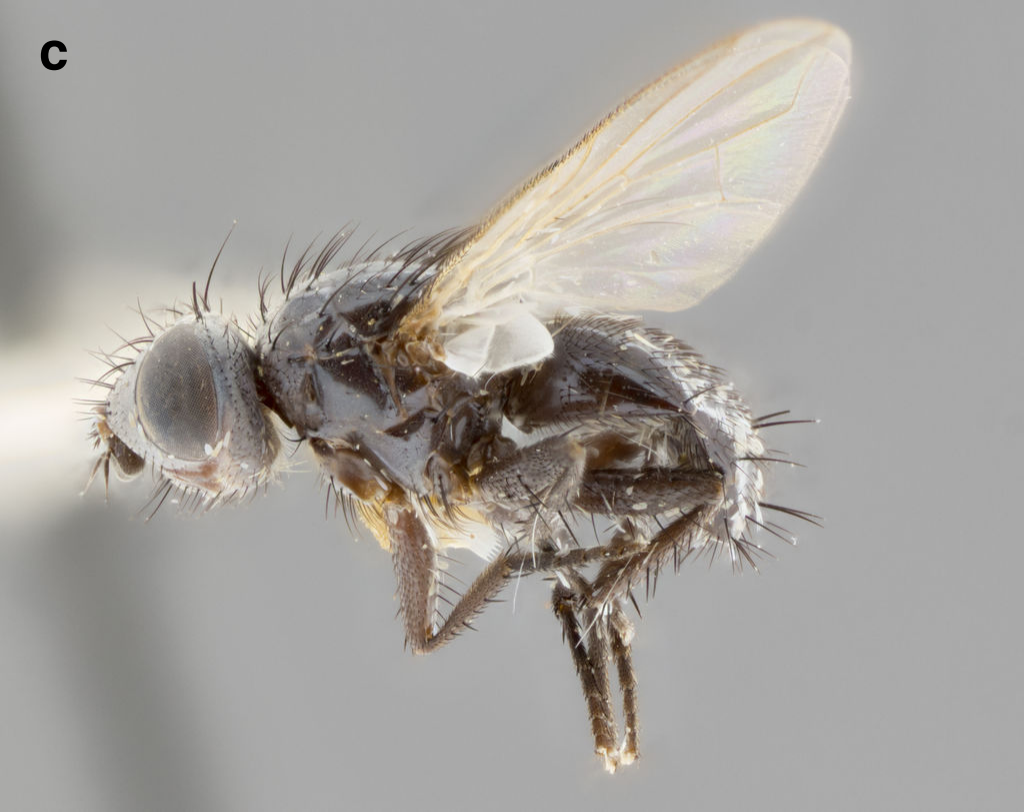
lateral view
